# Supplementary material for: Dioecy in a wind‐pollinated herb explained by disruptive selection on sex allocation via inbreeding avoidance
Source: New Phytol. 2025 Jul 6;247(6):2733–45. doi: 10.1111/nph.70356 (PMC12371145; doi:10.1111/nph.70356)
Supplement: Supplementary file 1 — Fig. S1 Variation of plant size and sex allocation in the study populations. Fig. S2 Plots showing the patterns of selection on gender via total fitness under two intermediate levels of inbreeding depression in plants of different sizes. Fig. S3 Histogram showing the size of plants in the three experimental populations. Fig. S4 Plots showing the nonlinear trade‐off curves between female and male functions in the studied populations. Fig. S5 Plot showing the effect of the number of male flowers on the selfing rate. Fig. S6 Plots showing the interactive effects of plant size, degree of inbreeding depression, and gender on relative total fitness in three experimental populations. Fig. S7 The effect sizes of explanatory factors on relative total fitness in three experimental populations. Fig. S8 The effect sizes of eight ancillary traits, size, gender, and inbreeding depression on relative male, female, and total fitness. Methods S1 Setup of the experimental populations. Methods S2 Detailed structure of each regression model used in this study. Table S1 Principal component analysis on eight ancillary traits and their correlation with gender. Table S2 Details of sex allocation, biomass, and paternity analyses of the three experimental populations. Table S3 Summary table of the general effects of male flower number, size, and population on female flower number. Table S4 Summary table of the general effects of size, gender, and population on the selfing rate. Table S5 Summary table of the general effects of size, gender, scenarios of inbreeding depression, and population on relative female, male, and total fitness. Please note: Wiley is not responsible for the content or functionality of any Supporting Information supplied by the authors. Any queries (other than missing material) should be directed to the New Phytologist Central Office. [file NPH-247-2733-s001.pdf]

New Phytologist Supporting Information

Article title: Dioecy in a wind-pollinated herb explained by disruptive selection on sex allocation via inbreeding avoidance

Authors: Kai-Hsiu Chen and John R. Pannell

Article acceptance date: 13 June 2025

Method S1. Around 225 seeds of *Mercurialis annua* were randomly sampled from the bulk-harvested seed pools of each of the three replicate populations of an ongoing experiment in which females had evolved substantial male-flower production after the experimental removal of males (Cossard et al., 2021; Gerchen et al., 2024). The seeds had been stored since their harvest in 2020 at 4 ºC. The sampled seeds were sown individually in 9 x 5 well trays in a greenhouse at the University of Lausanne in July 2022. After five weeks, 61 seedlings (green dots in the figure below) were randomly selected and replotted into pots with a diameter of 16 cm (empty circles in the figure below) and were arranged in a five-layered hexagon for each of the three experimental populations of this study (in total N = 183 seedlings were used for the three populations).

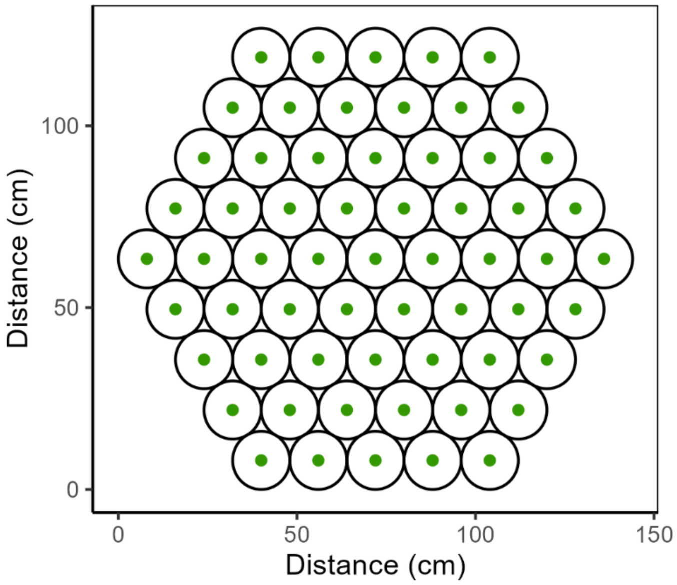

Method S2. Detailed structure of each regression model used in this study.

**Model aim**	**Response variable (model type, distribution)**	**Explanatory variables**	**Random variable**	**Sample size**	**Notes**
1. Trade-off	Female flower number (*glmmTMB*, negative-binomial)	Male flower number x Size x Population	Identity (to account for over-dispersion)	180 individuals	Zero-inflated model[1]
1. Selfing rate	Selfing rate (*glmer*, binomial)	Gender x Size x Population	Identity (to account for dispersion)	914 seeds from 173 individuals	Weighted by the number of paternity-assigned seeds
2. Fitness	Relative female fitness (*lmer*, Gaussian)	Gender x Size x δ x Population + Gender^2 x Size x δ x Population	Identity (to account for non-independency)	360 fitness estimates from 180 individuals	Variances weighted by plant size
	Relative male fitness (*lmer*, Gaussian)	Same as above	Same as above	Same as above	Same as above
	Relative total fitness (*lmer*, Gaussian)	Same as above	Same as above	Same as above	Same as above

Note: [1.] assuming that the probability of excess zeros in the response variable is the same across all levels of the predictor variables, regardless of their values (i.e., *zi* ~ -1).

Table S1. Rotated factor matrix for PCA (principal component analysis) on eight ancillary traits and its correlation with gender (male sex allocation) in the individuals of *M. annua* used in this study. The eight ancillary traits were the number of lateral branches, total length of lateral branches, mean length of lateral branches, number of leaves, total leaf area, mean leaf area, leaf dry biomass, leaf specific area sampled from the top 15 cm part of each individual (N = 175; 5 individuals with incomplete measurements of traits were not included). PCA axes showed no or weak (i.e., PCA2) correlation with sex allocation, indicating little or no sexual dimorphism in the study populations. See also Fig. **S8** for the effects of the ancillary traits on male, female, and total fitness.

	**Mean (SD)**	**PCA1**	**PCA2**	**PCA3**	**PCA4**	**PCA5**
Number of lateral branches	5.7 (2.5)	-0.10	-0.58	0.16	-0.64	0.05
Total length of lateral branches	21.2 (13.0) cm	-0.38	-0.40	-0.32	-0.11	0.33
Mean length of lateral branches	3.82 (2.19) cm	-0.36	-0.05	-0.52	0.40	0.33
Number of leaves	28.1 (14.6)	-0.38	-0.20	0.51	0.30	-0.01
Total leaf area	47.3 (34.2) cm^2	-0.50	0.21	0.20	-0.04	-0.17
Mean leaf area	1.82 (1.34) cm^2	-0.23	0.45	-0.39	-0.52	-0.20
Leaf dry biomass	160 (107) mg	-0.51	0.10	0.12	0.02	-0.37
Leaf specific area	0.29 (0.06) cm^2mg^{-1}	-0.10	0.44	0.37	-0.22	0.76
Cumulative variance explained (%)		0.41	0.63	0.79	0.89	0.98
Correlation coefficients with sex allocation (p-value)		0.07 (n.s.)	-0.21(p < 0.001)	0.001 (n.s.)	0.02 (n.s.)	0.05 (n.s.)

Table S2. Details of sex allocation, biomass, and paternity analyses of the three experimental populations. Population showed no difference in all the variables tested (*P* > 0.05; see the note below).

	**Population 1**	**Population 2**	**Population 3**
Sample size	60 (one individual dead)	60 (biomass for one individual missing and thus excluded)	60 (one individual dead)
Mean female flower number (SD)[1]	240 (200)	247 (362)	227 (267)
Mean male flower number (SD)[1]	340 (443)	276 (417)	334 (488)
Mean above-ground biomass (SD) (g) [2]	5.08 (2.36)	6.36 (5.78)	5.37 (4.35)
Mean mature seed number (SD)[2]	98.3 (91.7)	108 (201)	111 (169)
Total successfully genotyped seed number	319	314	315
Paternity assignment rate	94%	98%	97%
Average selfing rate[3]	30.2% (N = 60 individuals)	25.5% (N = 58 individuals) [4]	33.4% (N = 54 individuals) [4]
Mean outcrossing mating distance (cm)	28.6 (N = 210)	29.2 (N = 229)	27.8 (N = 215)
Maximum mating distance (cm)[5]	115 (N = 1)	115 (N = 1)	112 (N = 3)

Note: [1.] generalized model with negative binomial distribution; [2.] linear model with Gaussian distribution; [3.] generalized mixed model with binomial distribution and plant identity as a random factor; [4.] individuals producing no seed were excluded. [5.] Note that the possible maximum mating distance is 128 cm within the study populations.

Table S3. Summary table of the general effects of male flower number, size, and population on female flower number estimated by a generalized linear mixed model. The *P* values were extracted using likelihood ratio tests.

Notes: n.s. $P > 0.05$, ∗ $P < 0.05$, ∗∗ $P < 0.01$, ∗∗∗ $P < 0.001$

	**AIC**	**LRT**	***P* value**
Male flower	2228.4	47.71	***
Size	2326.4	145.69	***
Population	2180.0	1.29	n.s.
Male flower x Size	2176.0	16.94	***
Male flower x Population	2165.5	8.45	*
Size x Population	2157.1	0.05	n.s.
Male flower x Size x Population	2161.1	1.73	n.s.

Table S4. Summary table of the general effects of size, gender, and population on the selfing rate estimated by a generalized linear mixed model. The *P* values were extracted using likelihood ratio tests.

Notes: n.s. (non-significant) $P > 0.1$, m.n.s. (marginally non-significant) $P < 0.1$, ∗ $P < 0.05$, ∗∗ $P < 0.01$, ∗∗∗ $P < 0.001$

	**AIC**	**LRT**	***P* value**
Gender	566	30.5	***
Size	539	3.27	m.n.s. (*P* = 0.07)
Population	535	1.52	n.s.
Gender x Size	541	0.05	n.s.
Gender x Population	543	3.6	n.s.
Size x Population	540	0.37	n.s.
Gender x Size x Population	543	0.47	n.s.

Table S5. Summary table of the general effects of size, gender (linear and quadratic terms), scenarios of inbreeding depression (δ), and population on relative female, male, and total fitness estimated by linear mixed models.

Notes: n.s. $P > 0.05$, $* P < 0.05$, $** P < 0.01$, $*** P < 0.001$

	**Relative female fitness**		**Relative male fitness**		**Relative total fitness**	
	Sum sq.	*P* value	Sum sq.	*P* value	Sum sq.	*P* value
Size x Gender x δ x Population	0.01	n.s.	0	n.s.	0	n.s.
Size x Gender^2 x δ x Population	0.01	n.s.	0.01	n.s.	0.01	n.s.
Size x Gender x δ	0.38	***	0.07	n.s.	0.19	**
Size x Gender x Population	0.06	n.s.	0.03	n.s.	0.06	n.s.
Size x δ x Population	0.01	n.s.	0.01	n.s.	0	n.s.
Gender x δ x Population	0.03	n.s.	0.01	n.s.	0.02	n.s.
Size x Gender^2 x δ	0.27	**	0.26	**	0.27	***
Size x Gender^2 x Population	0.04	n.s.	0.05	n.s.	0.06	n.s.
Gender^2 x δ x Population	0.04	n.s.	0.02	n.s.	0.03	n.s.
Size x Gender	0.09	n.s.	0	n.s.	0.05	n.s.
Size x δ	0	n.s.	0.15	*	0.03	n.s.
Gender x δ	0.43	***	0.06	n.s.	0.2	**
Size x Population	0.13	n.s.	0.04	n.s.	0.11	n.s.
Gender x Population	0	n.s.	0	n.s.	0	n.s.
δ x Population	0.01	n.s.	0.01	n.s.	0.01	n.s.
Size x Gender^2	0.01	n.s.	0.09	n.s.	0.05	n.s.
δ x Gender^2	0.3	***	0.32	**	0.31	***
Population x Gender^2	0	n.s.	0	n.s.	0	n.s.
Size	1.58	***	1.23	***	2.05	***

Gender	0.24	**	0	n.s.	0.09	n.s.
δ	0	n.s.	0	n.s.	0	n.s.
Population	0.01	n.s.	0.01	n.s.	0.01	n.s.
$Gender^2$	0.04	n.s.	0.08	n.s.	0.08	n.s.

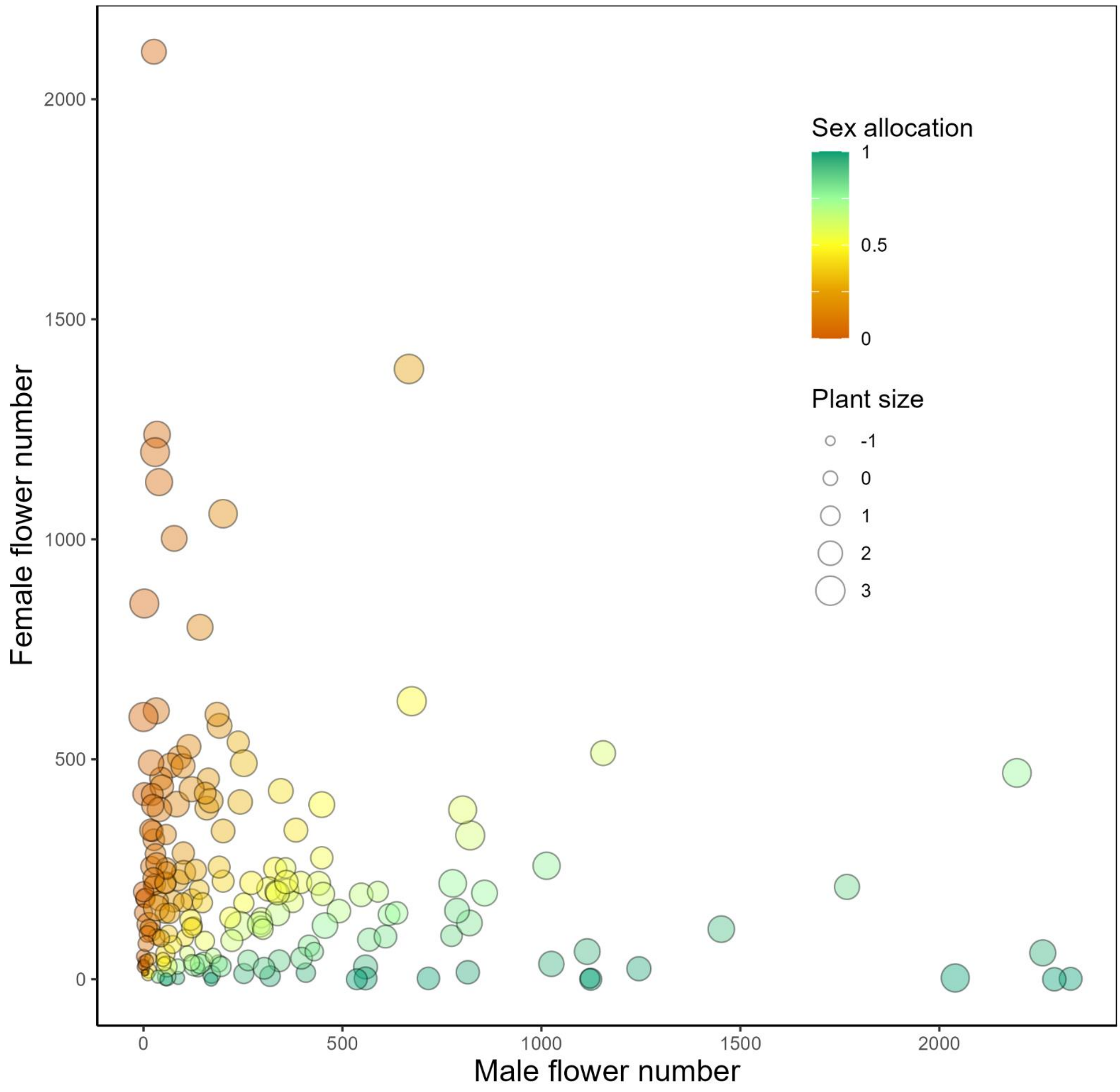

Figure S1. Variation of plant size and sex allocation in the study populations of *Mercurialis annua* (*N* = 180). Actual numbers of male and female flowers of each individual are shown with their sex allocation (maleness). The size of the points indicates the size of the plant. See the main text for details on the calculation of sex allocation and plant size.

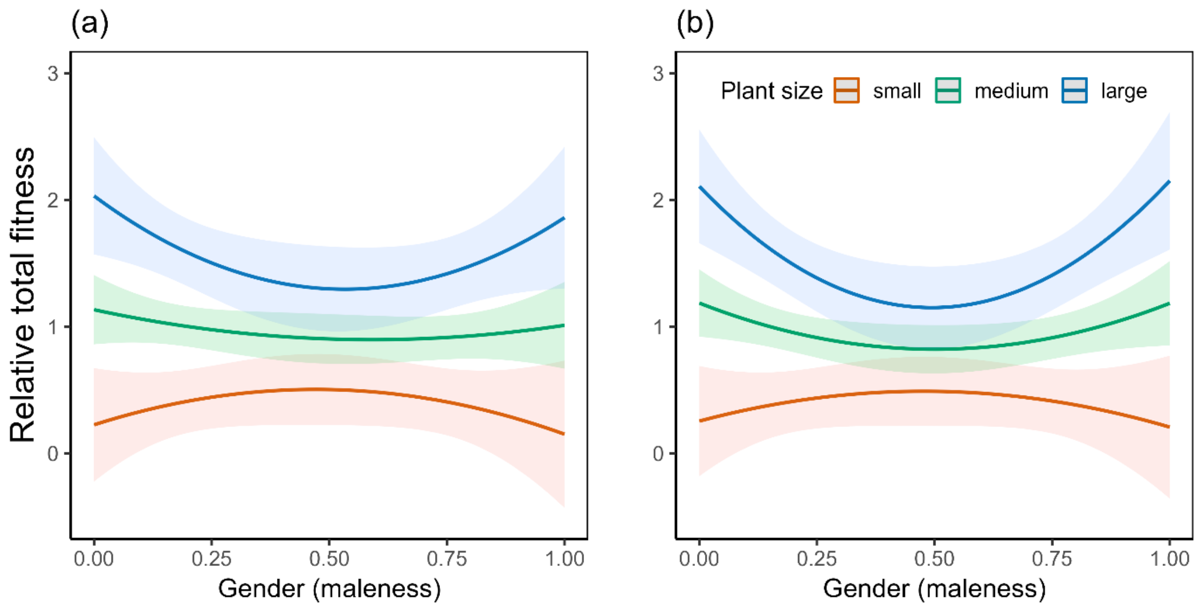

Figure S2. Plots showing the patterns of selection on gender via total fitness under two intermediate levels of inbreeding depression in plants of different sizes. (**a**) When inbreeding depression was 0.2, significant disruptive selection on gender was inferred for plants of large size (quadratic coefficient = 2.59 ± 1.25; $P < 0.05$), whereas no pattern of selection was inferred for plants of medium and small sizes ($P > 0.05$ for both linear and quadratic coefficients). (**b**) When inbreeding depression was 0.7, significant disruptive selection on gender was inferred for plants of medium and large sizes (quadratic coefficient = 1.43 ± 0.71 and 3.92 ± 1.22 with $P < 0.05$ and < 0.01, for medium and large plants, respectively), whereas no pattern of selection was inferred for plants of small size ($P > 0.05$ for both linear and quadratic coefficients). The shaded ribbons indicate the 95% confidence interval of the corresponding regression lines.

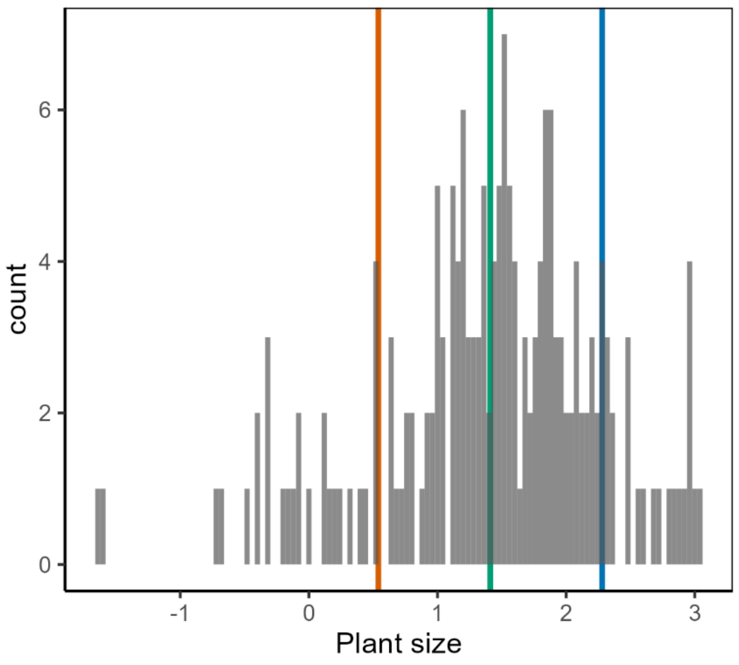

Figure S3. Histogram showing the size of plants in the three experimental populations of *Mercurialis annua* (N = 180). The mean size minus SD (at the 16% quantile; hereafter small plants), mean size (at the 50% quantile; hereafter medium plants), and mean size plus SD (at the 84% quantile; hereafter large plants) are indicated by orange, green, and blue vertical lines. Coefficients of an explanatory factor at the three levels of plant size were used to present the significant interactive effect of that factor with plant size on a response variable.

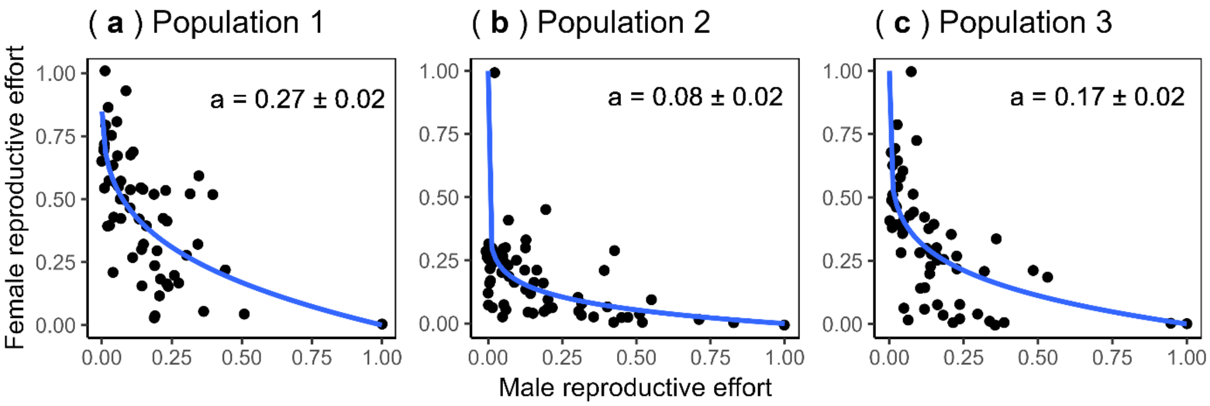

Figure S4. Plots showing the non-linear trade-off curves between female and male functions in studied populations. The non-linear curves were evaluated using non-linear least square regression (*nls* function in *nlme* package; Pinheiro et al., 2022) with the formula below. $RE_f = 1 - RE_m{}^a$, where RE_f and RE_m is the female and male reproductive efforts, respectively, defined as the number of flowers of that sex divided by the above-ground biomass of the plant and then relative to the plant with the highest effort in the population. The exponent *a* depicts the nonlinearity of the curves with $a < 1$, $= 1$, and > 1, indicating inward, linear, and outward trade-offs, respectively. The trade-off curves in the three populations were all concave, with the exponent *a* significantly smaller than one.

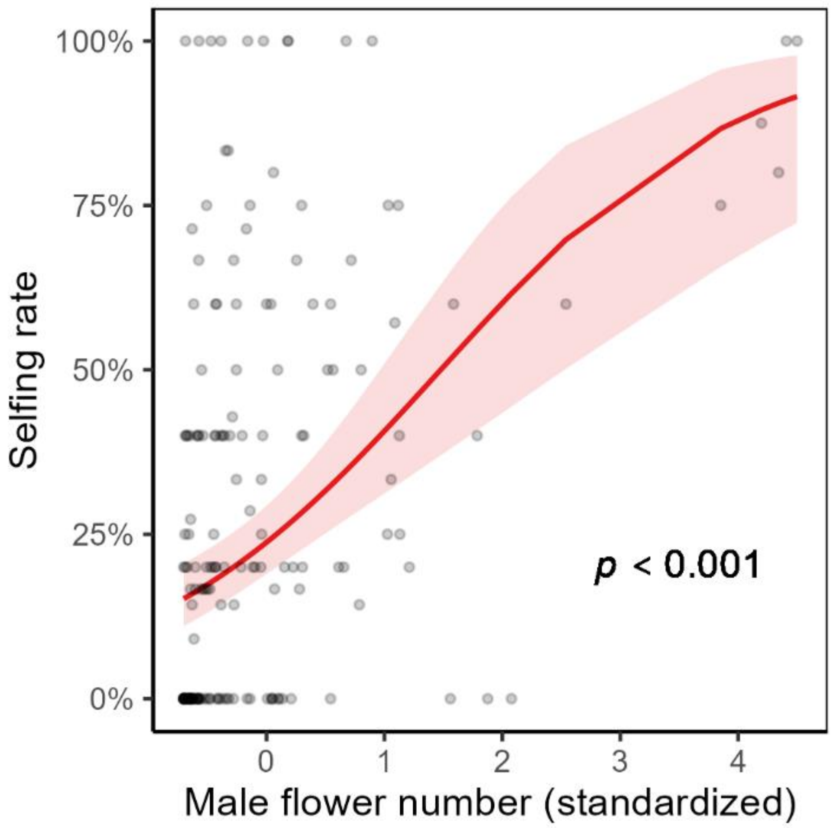

Figure S5. Plot showing the effect of the number of male flowers on the selfing rate in studied individuals (*N* = 172, individuals producing no mature seed were excluded). We used a generalized linear mixed model with a similar structure to the one presented in the main text (see Method S1) except that we replaced the explanatory variables of gender and size with male flower number here. The shaded ribbon indicates the 95% confidence interval of the regression lines.

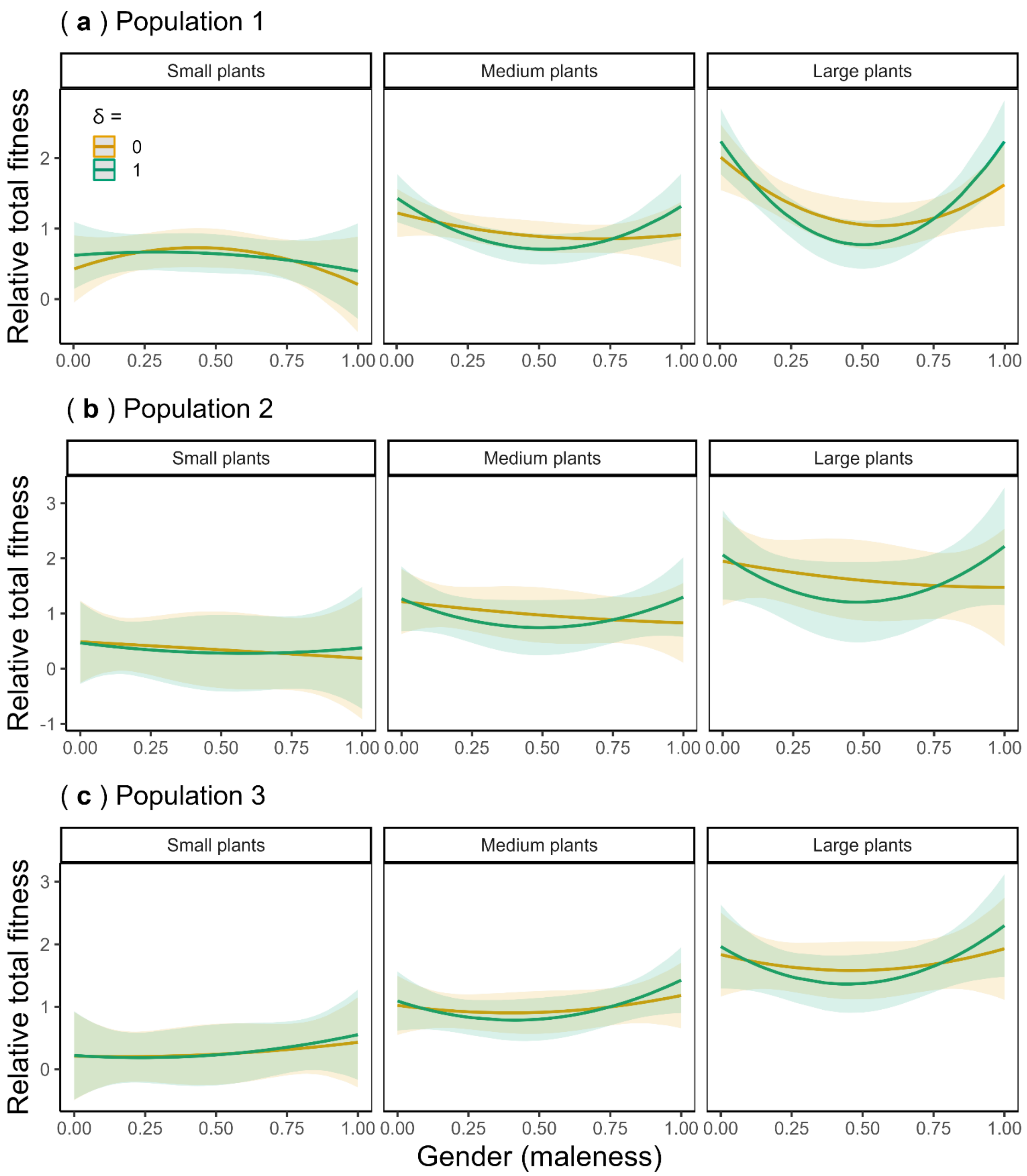

Figure S6. Plots showing the interactive effects of plant size, degree of inbreeding depression, and gender on relative total fitness of *Mercurialis annua* in three experimental populations analysed by three separated linear mixed models. Fitness was estimated under two scenarios of inbreeding depression (δ) of zero and one, depicted by orange and green lines, respectively. The shaded ribbons indicate the 95% confidence interval of the corresponding regression lines. See also Fig. **S7** for the effect sizes.

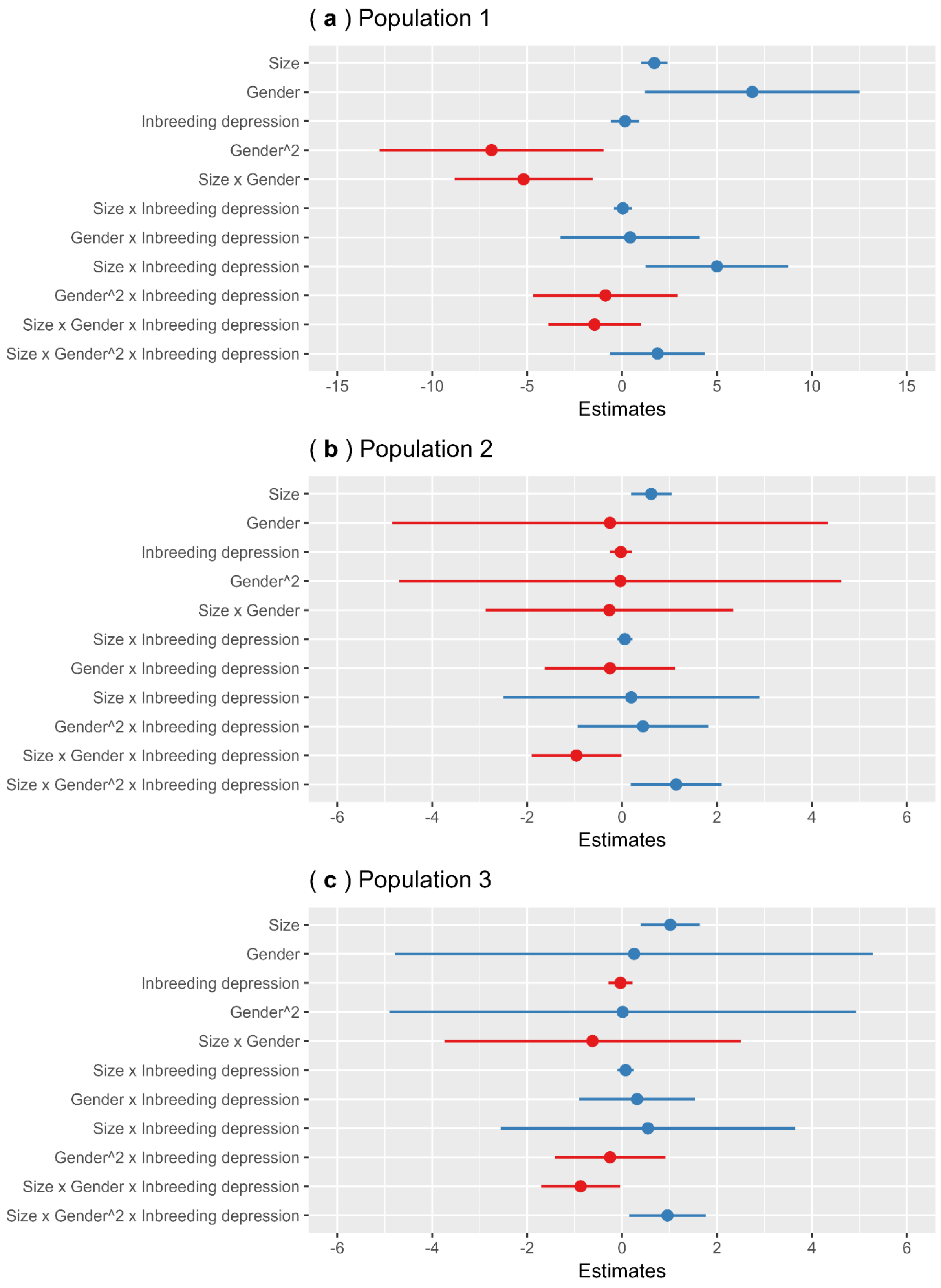

Figure S7. The effect sizes of explanatory factors on relative total fitness of *Mercurialis annua* in three experimental populations analysed by three separated linear mixed models (Fig. **S6**). Positive and negative effects are indicated by blue and red points, respectively.

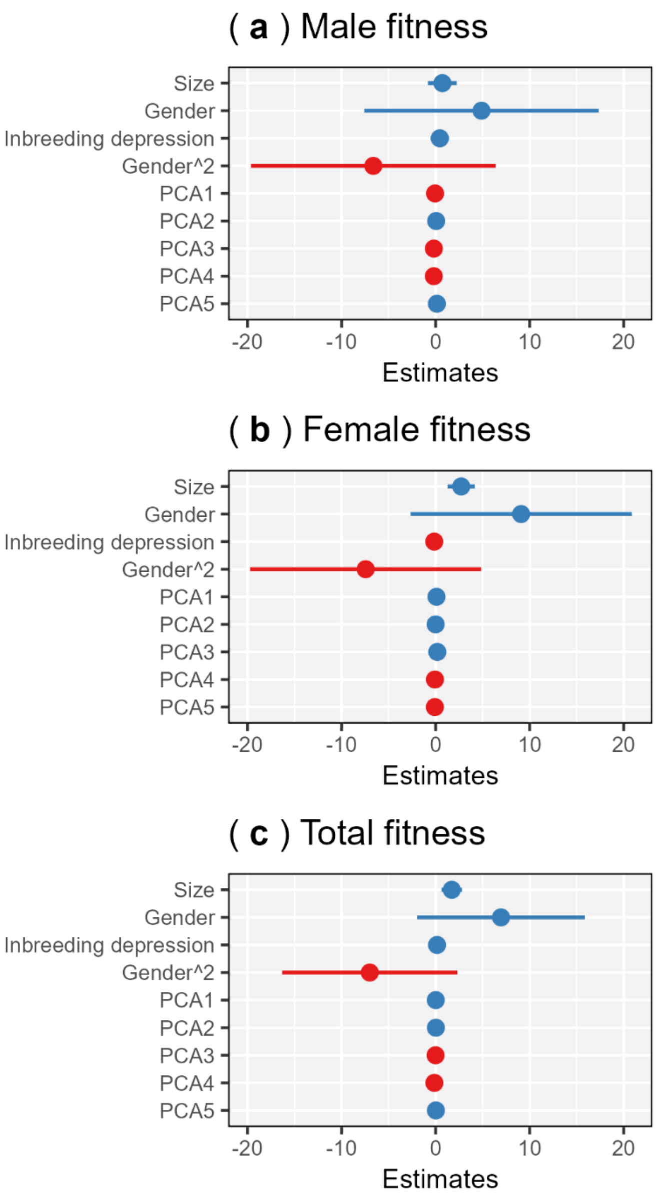

Figure S8. The effect sizes of eight ancillary traits (compressed into 5 PCA axes; see Table **S1**), size, gender (linear and quadratic terms), and inbreeding depression on relative male (**a**), female (**b**), and total (**c**) fitness estimated by linear mixed models (N = 175; five individuals with incomplete measurements of traits were not included). The PCA axes were added as single terms into the linear mixed models used for the analysis presented in the main text (see Method **S2** for their structures). For simplicity, only the effect sizes of a selected set of single terms were presented in the figures. The ancillary traits were thought to influence individual fitness via amelioration of pollen dispersal and/or pollen receipt. Nonetheless, according to the analyses, the ancillary traits likely played a minor role in determining individual fitness compared to sex allocation and size.

References

Pinheiro J, Bates D, DebRoy S, Sarkar D, R Core Team. **2022**. *nlme*: linear and nonlinear mixed effects models.
